# Beyond Lesions: Systemic Inflammatory and Metabolic Characterization of Swine Inflammation and Necrosis Syndrome

**DOI:** 10.3390/ani16172702

**Published:** 2026-08-31

**Authors:** Karien Koenders-van Gog, Eveline van Langen-Willems, Henriëtte Brouwer-Middelesch, Gerald Reiner

**Affiliations:** 1 Lintjeshof Veterinary Practice, LH Vet Group, 6031 RK Nederweert, The Netherlands; k.koenders@lintjeshof.com; 2 Merefelt Livestock Diagnostics, 6031 RK Nederweert, The Netherlands; 3 R&D Epidemiology, Royal GD, 7418 EZ Deventer, The Netherlands; e.willems@gddiergezondheid.nl (E.v.L.-W.); h.brouwer@gddiergezondheid.nl (H.B.-M.); 4 Clinic for Swine—Herd Health Management and Molecular Diagnostics, Justus-Liebig-University Giessen, 35392 Giessen, Germany

**Keywords:** animal welfare, tail lesions, hematology, clinical chemistry, pig, *Sus scrofa*

## Abstract

Swine Inflammation and Necrosis Syndrome (SINS) is characterized by multifocal inflammatory lesions in pigs of different ages and has been associated with systemic inflammatory processes. Therefore, the objective of the present study was to characterize associations between SINS expression and systemic inflammatory, hematological, and metabolic biomarkers in pigs from two farm cooperatives within the same commercial production system. In this exploratory study, 45 undocked piglets were assessed during the suckling and weaner stages. Clinical lesions were quantified across multiple body regions, and clinical-chemical and hematological parameters were analyzed in weaners. LASSO regression identified Pig major acute phase protein (PigMAP) as the biomarker most strongly associated with SINS-related traits, showing positive associations with tail base bristle loss and teat redness, whereas heel swelling and tail tip reddening were negatively associated or showed no association. Additional findings included decreased creatinine and urea under more favorable environmental conditions, and differences in bilirubin, creatine kinase (CK), lactate dehydrogenase (LDH), and glutamate dehydrogenase (GLDH) under less favorable conditions, indicating distinct patterns of clinical-chemical parameters associated with SINS. Platelet counts were lower in piglets with SINS status 2, i.e., piglets showing clinical signs of SINS during both the suckling and nursery periods, with statistically significant differences in the respective comparisons. These findings are consistent with the concept of SINS as a systemic inflammatory condition accompanied by measurable hematological and metabolic alterations. Together, these findings broaden the biological characterization of SINS and provide a foundation for future longitudinal studies investigating the temporal relationships between clinical manifestations and systemic biomarkers.

## Simple Summary

The swine inflammation and necrosis syndrome (SINS) affects pigs of different ages and is linked to welfare issues such as tail lesions. In a study of 45 weaned pigs with undocked tails from two farms, blood samples were analyzed for inflammatory markers (PigMAP), clinical chemistry (urea, bilirubin), and hematology (platelet count). Significant associations were identified between the clinical expression of SINS and several systemic biomarkers. These findings provide additional biological characterization of SINS and may contribute to a better understanding of the systemic inflammatory processes associated with its clinical expression.

## 1. Introduction

Swine Inflammation and Necrosis Syndrome (SINS) is a multifactorial condition characterized by inflammatory and necrotic lesions affecting peripheral body sites such as the tail base, tail tip, ears, teats, coronary bands, and heels [1]. These lesions can already be detected in suckling piglets [2] and may persist or reoccur after weaning [3], indicating that SINS is not restricted to a single production stage but develops early in life and may accompany animals throughout the production period [4,5]. Due to the association of tail lesions with pain, impaired welfare, carcass condemnation and potential long-term effects on robustness and performance [6,7,8,9,10,11,12,13,14,15,16], SINS has gained increasing attention in both experimental and applied pig research [17,18,19].

Although the clinical phenotype of SINS has been well characterized, considerably less is known about its systemic biological correlates. Accumulating evidence suggests that SINS is not merely the result of local mechanical damage or external irritation, but is associated with systemic pathological processes [2,20]. Previous studies have demonstrated associations between SINS and systemic inflammatory responses, including alterations in hematological parameters, acute phase proteins, and immune cell populations [21,22]. In particular, histopathological evidence of vasculitis, thrombosis, and inflammatory cell infiltration, together with alterations in monocyte populations, supports the concept that systemic inflammatory and microcirculatory disturbances contribute to lesion development [2,21,22]. Collectively, these findings support the concept that SINS is associated with systemic inflammatory processes rather than representing a purely local disorder.

Against this background, clinical-chemical and hematological parameters may provide important insights into the systemic manifestations associated with SINS. Several studies have reported associations between SINS and markers of inflammation, liver function, or metabolism, suggesting that inflammatory and metabolic pathways are involved in the syndrome [21,22,23]. However, these associations have been heterogeneous and appear to be influenced by factors such as age, husbandry conditions, genetic background, and the severity of clinical expression [4,5,21,22,23]. In particular, more pronounced or repeatedly occurring SINS phenotypes have been associated with more marked alterations in hematological and clinical-chemical parameters [22], whereas milder forms appear to be accompanied by more subtle systemic changes [4,5]. Despite these observations, the relationship between the clinical expression of SINS and systemic biomarker profiles under commercial production conditions remains incompletely characterized.

Acute phase proteins constitute an important link between local inflammatory processes and systemic inflammatory activation [24,25,26,27,28,29,30]. In pigs, positive acute phase proteins such as C-reactive protein (CRP), haptoglobin (Hp), serum amyloid A (SAA), and Pig major acute phase protein (PigMAP) increase rapidly in response to inflammatory or infectious stimuli and are regarded as sensitive indicators of systemic inflammation [31,32,33,34,35]. Among these proteins, CRP has repeatedly been associated with SINS severity [21,23], supporting the concept of systemic inflammatory involvement. However, CRP measurements are not consistently available across SINS studies, and other acute phase proteins have received considerably less attention.

PigMAP, also known as inter-alpha-trypsin inhibitor heavy chain 4 (ITIH4), is one of the major positive acute phase proteins in pigs. It is synthesized predominantly by hepatocytes in response to pro-inflammatory cytokines, particularly interleukin-6, and reflects generalized systemic inflammatory activation [33,36]. In addition, increased ITIH4 concentrations have been reported during bacterial bloodstream infections in humans, supporting its conserved role as a marker of systemic inflammation [37]. Despite its established value as a systemic inflammatory biomarker in pigs, PigMAP has not previously been investigated in relation to the clinical expression of SINS.

Interestingly, the gene encoding PigMAP (ITIH4) has previously been identified as a candidate gene associated with SINS, suggesting a potential biological link between this acute phase protein and the syndrome [38]. However, although ITIH4 was identified as a candidate gene, PigMAP itself has not previously been investigated as a phenotypic biomarker of SINS. Consequently, the potential relationship between genetic susceptibility and systemic PigMAP expression in SINS has remained unexplored.

Clinical-chemical, hematological, and acute-phase protein parameters provide complementary information on systemic inflammatory activation, metabolic status, and hepatic function and therefore represent suitable candidates for characterizing the systemic manifestations of SINS. Previous studies further suggest that the extent of systemic biomarker alterations varies with the clinical expression of the syndrome. Animals with more pronounced SINS phenotypes showed more marked hematological and clinical-chemical alterations [22], whereas populations with milder clinical manifestations exhibited only subtle systemic changes [4,5]. Such differences may additionally be influenced by farm-specific conditions, management, and genetic background [18,19]. Consequently, there is a need to further characterize systemic biomarker profiles associated with the clinical expression of SINS under commercial production conditions.

Therefore, the aim of the present study was to explore associations between the clinical expression of SINS and hematological, clinical-chemical, and acute phase protein biomarkers in pigs from two farm cooperatives within the same commercial production system. The aim was not to evaluate diagnostic thresholds but to identify biomarkers associated with variation in SINS severity under field conditions. Using an exploratory multivariable approach, we sought to identify the biomarkers most strongly associated with the clinical SINS phenotype and to evaluate whether these associations differed between farm cooperatives. We hypothesized that pigs differing in the clinical expression of SINS would also differ in their systemic inflammatory, hematological, and metabolic biomarker profiles measured at weaning.

## 2. Materials and Methods

### 2.1. Study Design

Data used in this study were derived from a longitudinal field investigation conducted within the framework of routine veterinary herd health assessments in two independent cooperatives belonging to the same Italian production integration between June 2024 and March 2025. The selected cooperatives offered the practical prerequisites required for the TAILSCAN project, including the availability of pigs with undocked tails, access to animals throughout production, and the feasibility of repeated clinical examinations and sampling under commercial conditions. The two cooperatives differed in several management and environmental characteristics (see Section 2.3), allowing the exploration of SINS-associated biomarker patterns in two farm cooperatives within the same commercial production system. The cooperatives differed with respect to health status, sow genetics, and nursery housing conditions. In contrast, feed composition and boar genetics were similar according to farm records and management information provided by the integration (see Section 2.3 for details). Each cooperative consisted of a sow herd, a nursery herd, and one or two finisher herds. Tail docking was not practiced. Piglets were weaned at four weeks of age and transferred to nursery units after weaning. All units were part of Italian Parma ham production. The two participating farms comprised approximately 1650 sows in total. For the underlying longitudinal clinical study, 44 sows and their litters were included, comprising 607 piglets examined during the suckling period. Of these, 596 piglets were still available for the second clinical SINS assessment after weaning.

Blood samples were initially collected from 46 randomly selected nursery piglets originating from 25 of the 44 previously examined litters. No selection based on clinical SINS findings, health status, sex, body weight, or any other phenotypic criterion was performed. Blood sampling and laboratory analyses were performed within the framework of routine veterinary diagnostic investigations. Consequently, only a subset of nursery piglets could be included in the hematological and biochemical analyses. One blood sample had to be excluded because coagulation prevented reliable laboratory analysis. Consequently, hematological and biochemical analyses were performed on 45 piglets.

**Figure 1:**
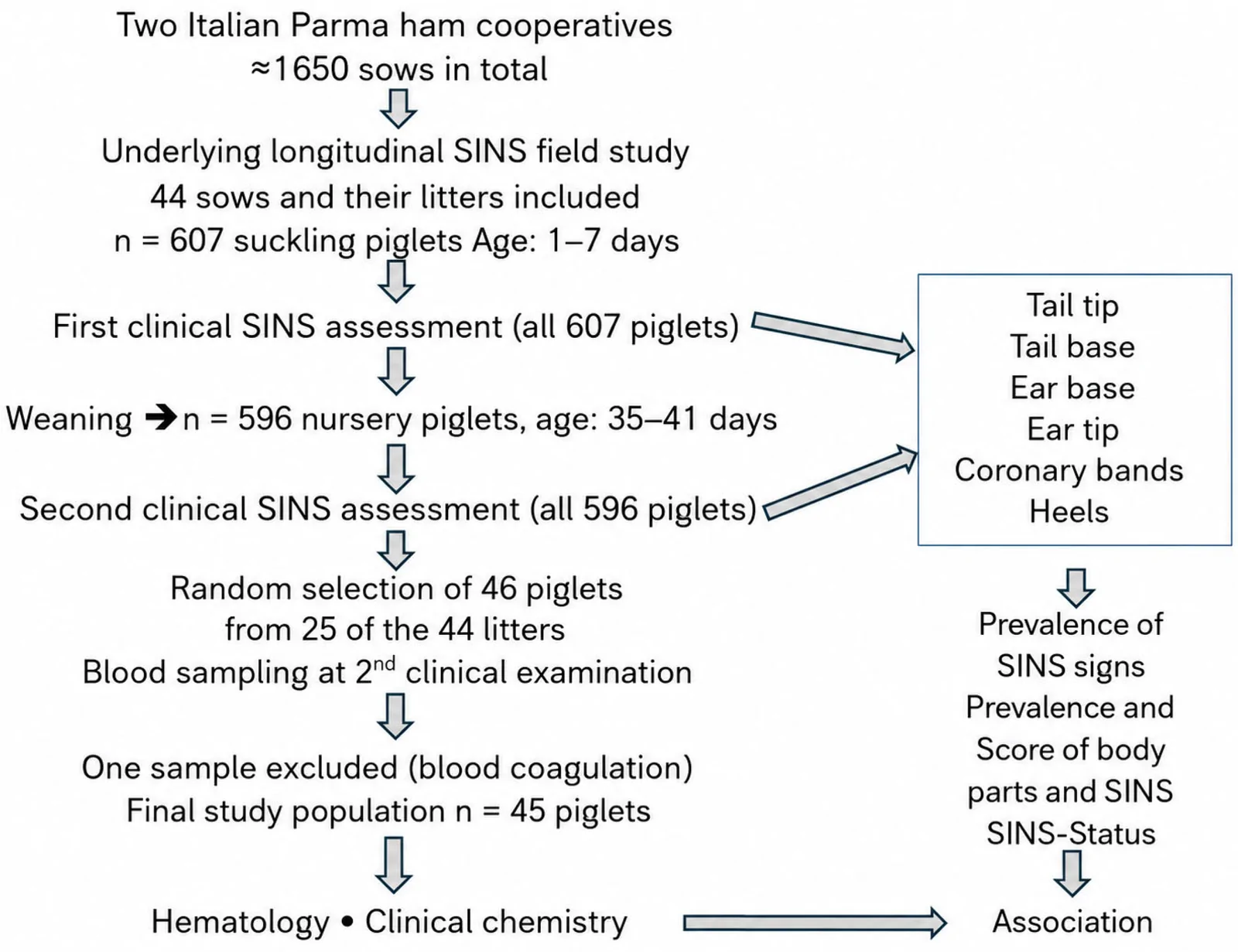
Study design. Flow chart illustrating the selection of animals, the two clinical SINS assessments during the suckling and nursery periods, random selection of piglets for blood sampling, and the final study population included in the hematological and clinical chemistry analyses.

Swine inflammation and necrosis syndrome (SINS) was recorded in suckling piglets and weaners at 1–7 days and 35–41 days of age, respectively. The time points of animal assessment are visualized in Figure 1.

Blood samples were taken at the moment of the clinical assessment as weaners (days 35 to 41). Samples were analysed by PCR as part of the routine herd health monitoring program. Blood samples were pooled according to standard veterinary surveillance procedures (five samples per pool). All pools tested negative for PRRSV. For PCV2, 2 of 10 pools (both from cooperative 1) tested positive by quantitative PCR, but only at very low viral loads (<2 × 10^1^ genome equivalents per µL). To limit invasive procedures, the same blood samples were used for additional analysis on biomarkers for welfare.

### 2.2. Animal Assessment

Piglets were individually identified using LeeO RFID ear tags (LeeO Precision Farming, Deventer, The Netherlands), enabling digital animal tracking throughout the study. Piglets were clinically assessed twice during the study: once during the suckling period (1–7 days of age) and once during the nursery phase (35–41 days of age). Each animal was examined only once within each production stage. Blood samples for hematological and clinical–chemical analyses were obtained immediately prior to the nursery-phase examination. Clinical assessments were performed jointly by two trained investigators, and lesion scores were assigned by consensus during the examination (Table 1). As all animals were evaluated by the same investigators using the same scoring criteria, variability due to different examiners was avoided. The following clinical parameters were assessed in neonatal and weaned pigs and recorded as present or absent as described in detail by [5] (see Table 1). The SINS scoring system applied in the present study was based on previously published SINS assessment protocols [1,2,3,4,5,17,18,19,20,21,22,23]. As these protocols differ slightly with respect to the evaluated body regions and lesion types, the scoring system was adapted to the objectives of the present study and to the practical requirements of repeated clinical examinations under commercial field conditions.

For each piglet, all clinical findings listed in Table 1 were assessed separately during the suckling and nursery periods and scored binarily as absent (0) or present (1). The individual lesion scores were summed to obtain body-part scores (e.g., for the tail base) and, across all body parts, a SINS score for each examination, with possible values ranging from 0 (no clinical signs) to 24 (all recorded lesions present). For each body region, prevalence was calculated as the proportion of piglets with a body-part score greater than 0. Likewise, the prevalence of SINS was calculated as the proportion of piglets with a SINS score greater than 0.

Based on the SINS scores obtained during the suckling and nursery periods, each piglet was assigned a longitudinal SINS status. SINS status 0 was assigned to piglets with a SINS score of 0 at both examinations. SINS status 1 was assigned to piglets with a SINS score of 0 at either the suckling or the nursery examination. SINS status 2 was assigned to piglets with a SINS score greater than 0 at both examinations. The same procedure was applied to classify the longitudinal tail base status.

### 2.3. Farm Characteristics

#### 2.3.1. Sow Farm 1

Sow Farm 1 housed approximately 750 PIC sows (PIC, Diessen, The Netherlands) and used Fomeva (Topigs Norsvin Italia S.r.l., Manerbio, Italy) (parity 1) and Goland (Gorzagri S.S., Fonzaso, Italy) (parity > 1) boars for artificial insemination. Repeated PCR testing of processing fluids remained negative for PRRSV throughout the observation period.

Replacement gilts were vaccinated against PRRSV using a modified live vaccine. Piglets were not vaccinated against PRRSV but received routine vaccinations against PCV2 and *Mesomycoplasma hyopneumoniae*. According to routine herd monitoring, repeated PCR testing of processing fluids remained negative for PRRSV throughout the observation period, consistent with the classification of a stable breeding herd according to current PRRSV monitoring systems.

Lactating sows were housed in crates and fed ad libitum with dry meal feed supplied manually at least four times daily. Sows had nipple drinkers and received additional water via a trough with a tap every morning, with an average intake of 26 L/day. Piglets had nipple drinkers and additional water in bowls. Enrichment consisted of chains and nesting material made of paper. Climate conditions were favorable, with CO_2_ concentrations ranging from 450 to 950 ppm, determined by routine measurements using a portable CO_2_ meter. Incoming air was cooled by a water system. Flooring in the farrowing unit consisted of plastic slats, with cast iron slats under the sow, and a piglet nest was located at the head side.

Gestating sows were housed in groups and had ad libitum access to water via easily accessible drinkers. Mean water consumption was approximately 13–17 L per sow per day. According to farm records, no toxin binders were included in the feed. Feed was provided in two phases during gestation. During the first weeks, sows were housed in crates. Piglets were weaned at four weeks of age.

Nursery 1 was straw-bedded, housing 150 pigs per pen, with 10 nipple drinkers and 15 feeding places. Pens were equipped with 10 nipple drinkers for approximately 150 pigs, corresponding to one drinker per 15 pigs. Piglets were fed meal for the first two weeks post-weaning, then switched to pelleted feed. Nursery stay lasted until 12 weeks of age.

#### 2.3.2. Sow Farm 2

Sow Farm 2 housed *n* = 900 sows of Topigs genetics (Topigs Norsvin, ‘s-Hertogenbosch, The Netherlands), with Fomeva boar genetics. The farm operated a three-week batch farrowing system. A PRRSV field strain infection was present, and a PRRSV vaccination program was implemented on the farm. Clinical signs of arthritis were observed in suckling piglets during the study period. Piglets were vaccinated against PCV2 and *Mesomycoplasma hyopneumoniae* but not against PRRSV.

Gestating sows were housed in groups of 98 individuals, fed via electronic feeding stations, and had 8–10 drinkers per group. Water intake was not measured. Enrichment included chains with wooden blocks and occasional straw/hay.

Lactating sows were housed in crates with plastic slatted floors and fed twice daily with pelleted feed via a valve-operated trough. Climate conditions were characterized by an ambient temperature of approximately 29 °C, which is above the thermoneutral range generally recommended for lactating sows, and by CO_2_ concentrations below 3000 ppm. Sows and piglets had access to nipple drinkers. No nesting material or additional water sources were provided. Enrichment consisted of a movable tool attached to the crate.

Nursery 2 featured concrete flooring, 80 pigs per pen, 10 feeding places, 5 nipple drinkers, and 2 open water troughs per pen. Enrichment consisted of ropes. At the time of assessment, pigs showed clinical signs consistent with weaning diarrhea and respiratory disease, including coughing.

### 2.4. Blood Analysis

Blood samples for hematological and clinical–chemical analyses were collected via venipuncture immediately before the clinical examination during the nursery phase (age 35 to 41 days). Sampling was performed within the framework of a routine veterinary herd health investigation and not solely for experimental purposes. One sample was allowed to clot at room temperature before centrifugation (10 min at approximately 2000–3000× *g*). Serum was separated and stored at −20 °C until analysis. Samples were analyzed at the veterinary laboratory of Royal GD (Deventer, The Netherlands) using a DxC 700 AU Chemistry Analyzer (Beckman Coulter, Woerden, The Netherlands). A second sample was stabilized with EDTA, cooled to 4 °C and processed within 24 h. Hematology was performed at Merefelt Livestock Diagnostics (part of Lintjeshof LH Vet group) with Idexx Pro Cyte DX (Idexx Laboratories, Hoofddorp, The Netherlands).

All analyses were performed according to the laboratory’s standard operating procedures under a certified quality management system (NEN-EN-ISO 9001:2015) [39], including routine calibration and internal quality control using commercial control sera. Analytical methods applied in this study are widely established in veterinary clinical chemistry and have been validated or verified for use in porcine samples [40,41].

Colorimetric methods were used to determine serum calcium, phosphate (ammonium molybdate method), magnesium, total protein (biuret method), albumin (bromocresol green method), and total bilirubin concentrations (Beckman Coulter reagents; Beckman Coulter, Woerden, The Netherlands). Haptoglobin concentrations were measured using a colorimetric assay (PHASE Haptoglobin Assay, Tridelta Development Ltd.; Sagittarius Science, Bunde, The Netherlands), and pig major acute-phase protein (PigMAP) was determined using a turbidimetric assay (Turbovet pig-MAP, Acuvet Biotech, Zaragoza, Spain). Selection and biological interpretation of the acute-phase proteins were based on previous studies in pigs [28,30,42], which identified these proteins as sensitive markers of systemic inflammatory responses. No diagnostic threshold values were applied; instead, associations between protein concentrations and SINS-related traits were evaluated statistically. Enzymatic methods were applied for the determination of urea (urease method), creatinine, and non-esterified fatty acids (NEFA; Wako NEFA-HR(2), Fujifilm Wako Chemicals, Neuss, Germany). Activities of CPK, GGT, ALT, LDH, ALP, and AST were measured according to IFCC reference procedures [43] using Beckman Coulter reagents. GLDH activity was determined according to DGKC recommendations (Roche Diagnostics, Almere, The Netherlands). Published reference values and laboratory-specific reference intervals for pigs [44] were used to provide biological context. However, the analyses focused on associations between biomarker concentrations and SINS-related traits rather than on deviations from reference ranges.

### 2.5. Statistics

Data were analyzed using IBM SPSS Statistics, version 29 (IBM Corp., Munich, Germany). Owing to the limited sample size and pronounced differences between farm cooperations (coops), the degrees of freedom did not allow the inclusion of additional minor factors in the statistical models. Piglets originated from 25 different sows, with most sows contributing only one or two piglets to the study. Because the number of observations within most sows was too small to provide substantial information for estimating between-sow variance, and given the limited overall sample size, sow was not included as a random effect. Marked coop-specific differences were observed in the associations between SINS traits and clinical-chemical or hematological parameters, resulting in frequent SINS × coop interactions. Because these interactions indicated that the relationships between biomarkers and SINS differed substantially between coops, analyses were performed separately for each coop rather than fitting a single pooled model. As this study was designed as a pilot study with an exploratory, hypothesis-generating character, no correction for multiple testing (e.g., Bonferroni adjustment) was applied. This approach was chosen to avoid overlooking potentially biologically relevant associations due to overly conservative alpha error control. Accordingly, results should be interpreted as exploratory.

The prevalences and scores of SINS lesions were analysed descriptively, using generalised linear models (GLM) adapted to binary or metric response variables, as appropriate. Suckling piglets and weaners were analysed separately. Sex was included as a fixed effect, and age (in days) was included as a covariate.

To evaluate overall differences in clinical–chemical and hematological parameters between the two cooperatives, generalized linear models including cooperative, sex, and age were fitted. These analyses revealed pronounced cooperative effects for several variables. In addition, exploratory analyses investigating associations between SINS traits and biomarker concentrations identified frequent and substantial SINS × cooperative interactions, indicating that the relationships between SINS and the measured parameters differed between cooperatives. Consequently, the associations between SINS and clinical–chemical or hematological parameters were analysed separately for each cooperative in all subsequent analyses. In a first step, a Least Absolute Shrinkage and Selection Operator (LASSO) regression screening was performed to identify, from the large number of candidate variables, those showing the strongest associations with the clinical expression of SINS. The LASSO regression is particularly suited for variable selection in high-dimensional datasets, as it penalises regression coefficients and shrinks non-informative predictors towards zero [45].

In a second step, variables selected by LASSO were further evaluated using generalised linear models, including SINS status and sex as fixed effects and age as a covariate, to assess their statistical significance and biological relevance.

An analogous modelling approach was applied using the status of SINS lesions at the tail base instead of the SINS status. Tail base lesions were analysed separately because this lesion site showed significant associations with several biomarkers in the present study and has repeatedly been identified as a prominent manifestation of SINS in previous investigations (e.g., [4,21,22]).

## 3. Results

### 3.1. Effect of the Farm on Clinical Chemical/Hematological Parameters

A total of 45 piglets were included in the analysis. For all animals, data were available from both the suckling and the weaning period. The prevalences of inflammatory and necrotic lesions at the examined body sites, as well as the prevalence of SINS (Score > 0), are summarised in Table 2. Several SINS-related traits showed differing distributions between the two cooperatives. Given the limited sample size and exploratory design of the study, statistical evidence for these differences was generally weak, with heel alterations in suckling piglets representing the only trait that differed significantly between cooperatives.

In suckling piglets, lesions of the tail base and coronary bands were most prevalent, whereas in weaners the tail base was the most frequently affected site. In cooperative 2, heel alterations were also common, affecting 54% of the piglets. Overall, the variability of the recorded traits was high. No effects of sex were detected. Piglet age (days) had a significant effect on tail base lesions in both age groups and on heel lesions in suckling piglets.

Most analysed clinical chemistry and hematological parameters also showed considerable variability. No effects of sex or age were detected. Significant farm effects were observed for alkaline phosphatase (ALP), gamma-glutamyl transferase (GGT), urea, calcium, white blood cell counts, and monocyte concentrations (Table 3). The majority of values were within the reference ranges considered physiological for this age group. Nevertheless, continuous variation within these physiological ranges was further analysed because the objective of the study was to identify associations between biomarker profiles and the clinical expression of SINS rather than to classify pigs according to diagnostic reference limits.

### 3.2. Associations of SINS and SINS Signs at the Tail Base with Clinical Chemical/Hematological Parameters

Multiple LASSO regression was performed to screen for associations between SINS scores and clinical-chemical as well as hematological parameters (Table 4). As no correction for multiple testing was applied in this exploratory analysis, the *p*-values reported for these associations are nominal and should be interpreted cautiously and considered to require independent confirmation. Different association patterns emerged between cooperative 1 and cooperative 2.

In cooperative 1, PigMAP was consistently selected as the strongest positive predictor of SINS in both suckling piglets and weaners. Higher SINS scores were additionally associated with lower creatinine and urea concentrations and higher calcium concentrations. Subsequent generalized linear models confirmed significant associations of PigMAP and creatinine with SINS in both production stages.

In cooperative 2, haptoglobin was selected as a positive predictor of SINS in suckling piglets, whereas bilirubin, calcium, CK, LDH, and GLDH showed negative associations. Subsequent generalized linear models identified bilirubin as the only parameter that remained significantly associated with SINS.

With regard to SINS-related tail base lesions (Table 5), LASSO identified an association pattern in cooperative 1 that closely resembled that observed for the SINS score. In cooperative 2, an additional positive association with PigMAP was identified. The results were further evaluated using generalized linear models. Analyses were performed separately for each cooperative and compared the distributions of piglets that were free of SINS both as suckling piglets and as weaners (Sins status 0), piglets showing SINS signs either as suckling piglets or as weaners (SINS status 1), and piglets showing SINS signs both as suckling piglets and as weaners (SINS status 2) (Table 6). Across both cooperatives, 10 piglets were classified as SINS status 0, 12 as SINS status 1, and 23 as SINS status 2. Of the 12 piglets with SINS status 1, 10 were SINS-positive only during the suckling period and 2 only during the nursery period.

In cooperative 1, exploratory analyses identified significant associations between SINS score and PigMAP, urea, creatinine, and calcium. In addition, a significant association with alkaline phosphatase (ALP) was detected. In cooperative 2, significant associations were observed for bilirubin and platelet counts.

**Figure 2:**
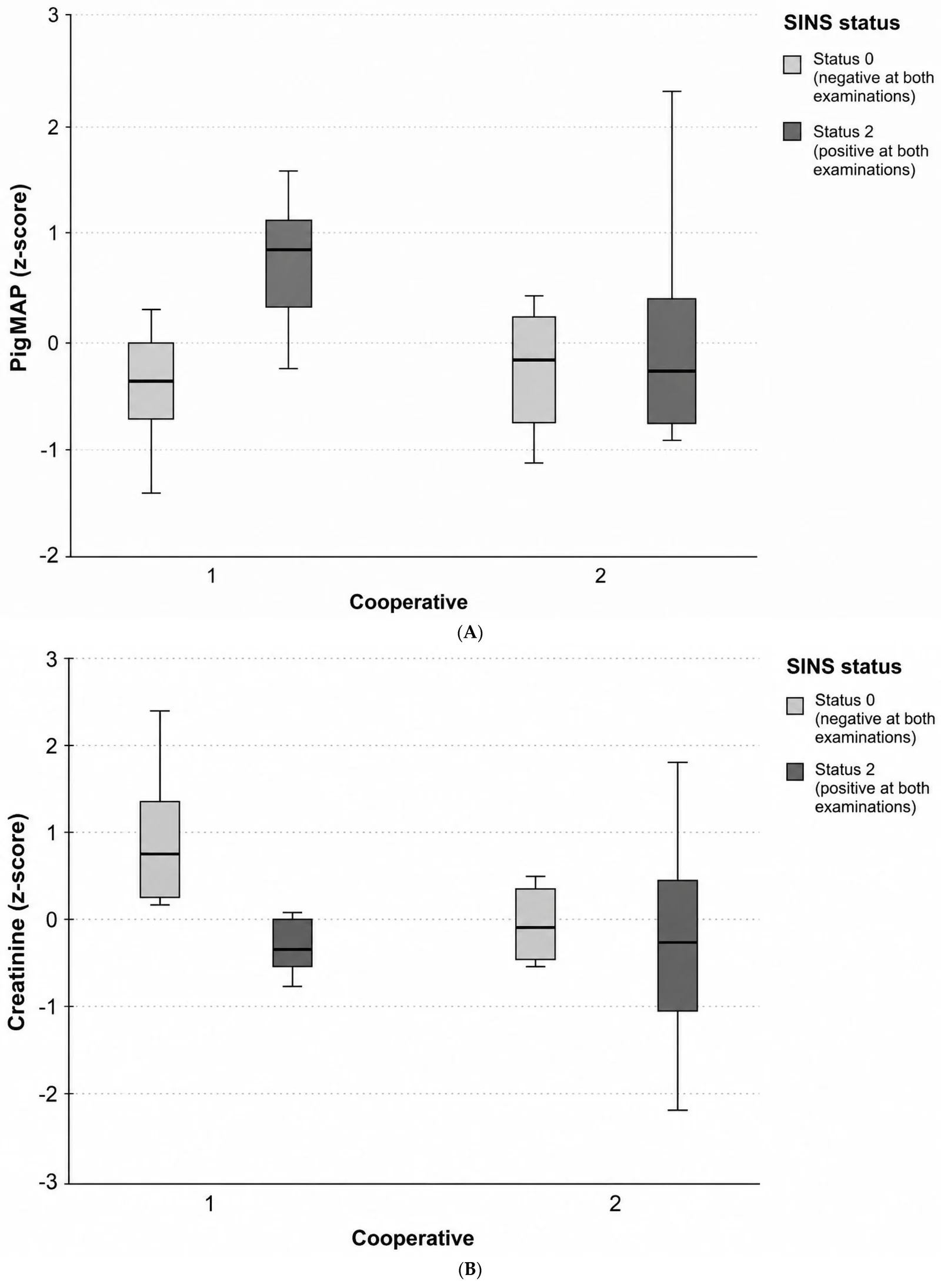

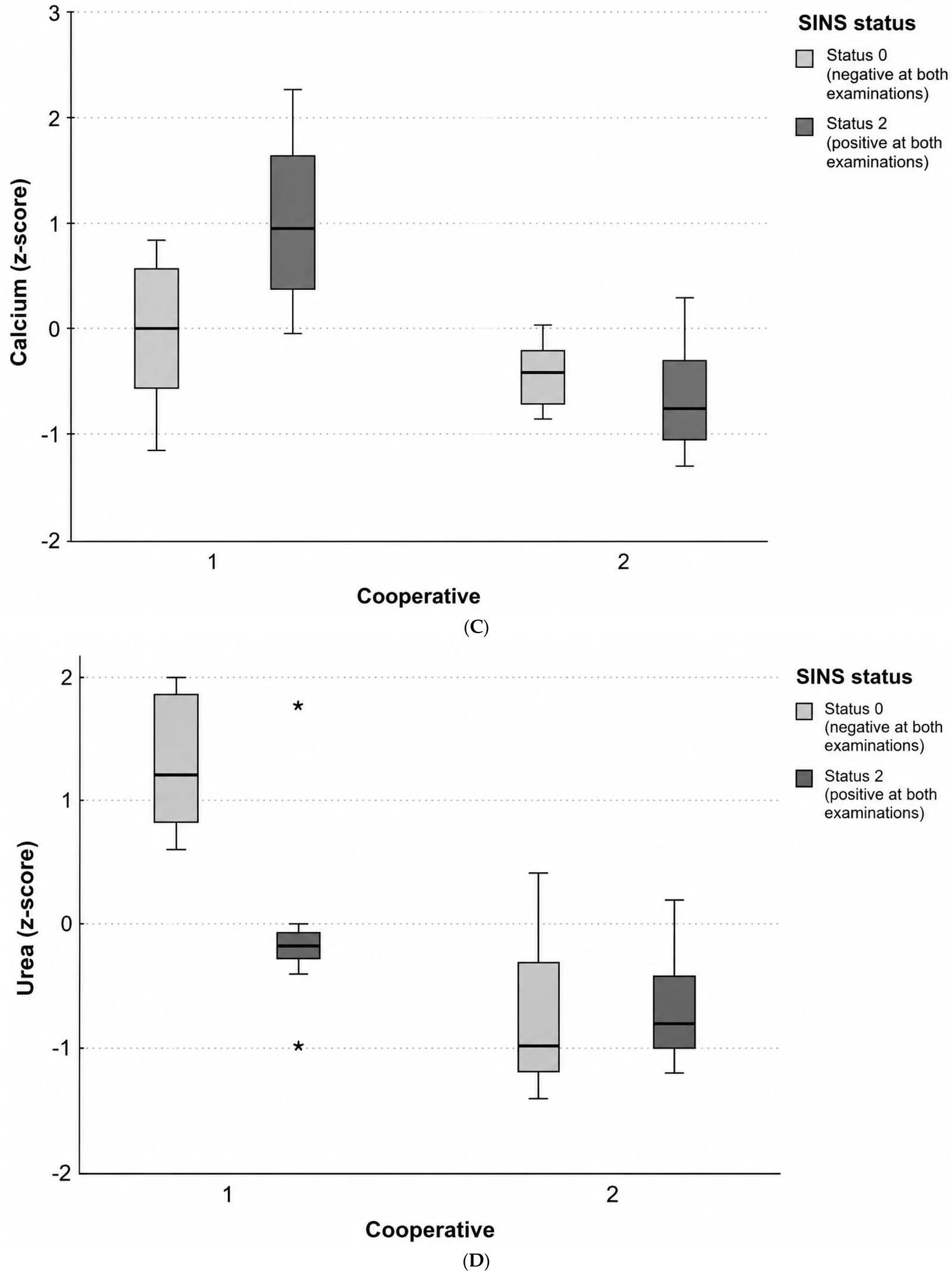

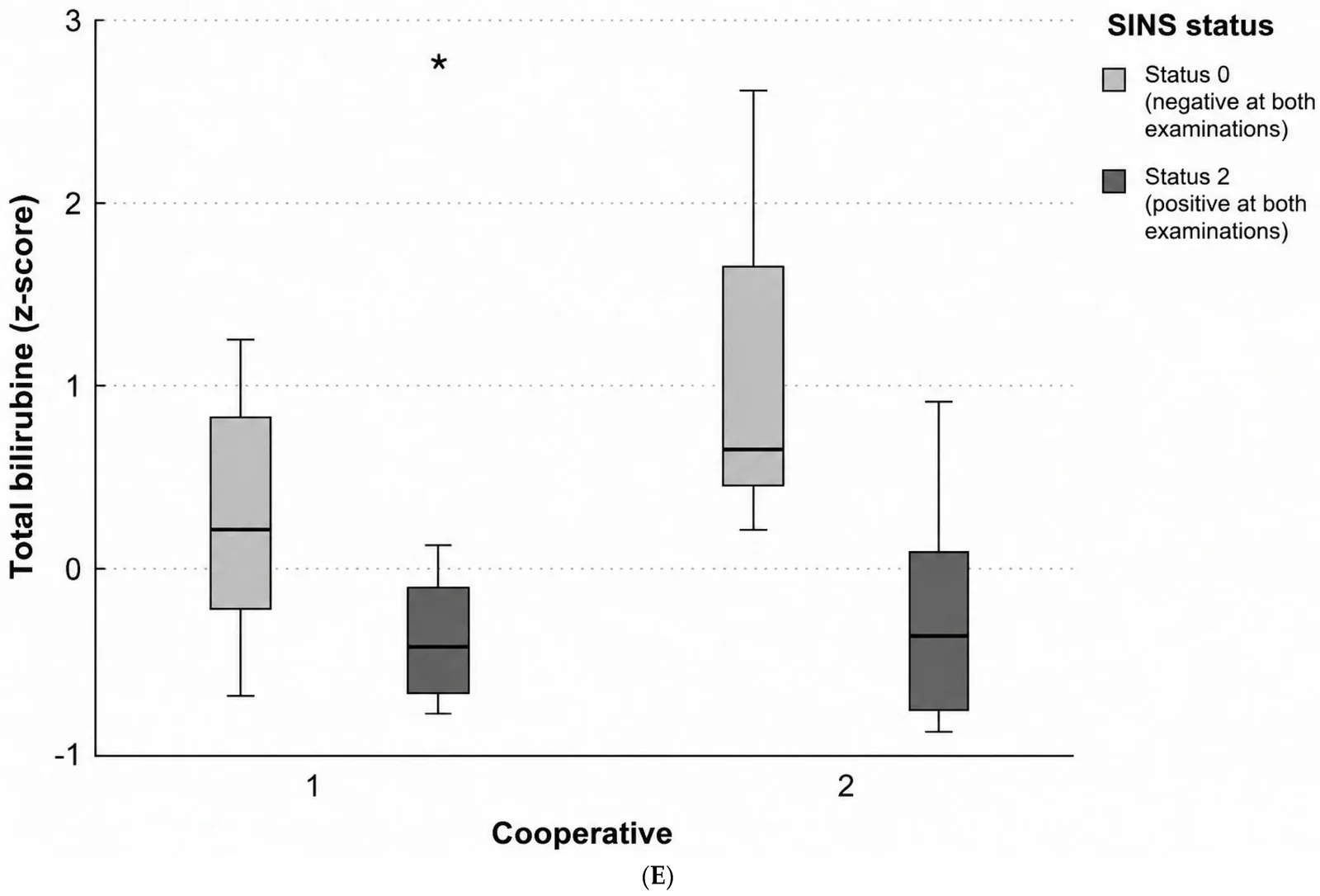
Associations between SINS and selected clinical-chemical parameters (Z-transformed) by farm (cooperative 1 and 2), including PigMAP (**A**), creatinine (**B**), calcium (**C**), urea (**D**), and bilirubin (**E**). The black line indicates the median, boxes represent the interquartile range (50% of piglets), and whiskers represent the 5th–95th percentiles (90% of piglets). Light grey boxes denote piglets with SINS status 0 (SINS score = 0 both as suckling piglets and as weaners), while dark grey boxes denote piglets with SINS status 2 (SINS score > 0 both as suckling piglets and as weaners). SINS status 1 animals (SINS-positive at only one of the two examinations) were omitted from the visualisation to allow a direct comparison between consistently SINS-negative and consistently SINS-positive piglets. Significant associations were observed for PigMAP, creatinine, calcium, and urea in cooperative 1 (*p* < 0.05) and for bilirubin in cooperative 2 (*p* < 0.05). Asterisks (*) indicate outliers.

Figure 2 illustrates the associations between piglets with SINS status 0 and SINS status 2 and selected clinical-chemical parameters, including PigMAP (Figure 2A), creatinine (Figure 2B), calcium (Figure 2C), urea (Figure 2D), and bilirubin (Figure 2E), separated by farm cooperative. The figure supports the results presented in Table 5. In cooperative 1, a higher SINS status was associated with increased PigMAP and calcium levels, as well as with decreased creatinine and urea concentrations. In cooperative 2, bilirubin showed a significant negative association with the SINS status. These patterns visually illustrate the differential associations observed between the two cooperatives.

## 4. Discussion

### 4.1. Systemic Biomarker Profiles Associated with the Clinical Expression of SINS

The objective of the present study was not to diagnose systemic disease based on abnormal laboratory values or to determine the proportion of pigs outside physiological reference ranges. Instead, we investigated whether variation in hematological and biochemical biomarkers was associated with variation in the clinical expression of SINS. Consequently, biomarkers were analysed as continuous variables, because biologically meaningful associations may occur within physiological reference intervals.

This distinction is important because population-based reference intervals are primarily intended to distinguish overt disease from health in individual animals and to support clinical decision-making. They are not designed to detect subtle biological variation associated with multifactorial disease processes or differences in disease severity within clinically comparable populations. Modern laboratory medicine increasingly recognizes that individual organisms usually fluctuate within a substantially narrower physiological range than the population as a whole. Consequently, statistically significant associations between continuous biomarker concentrations and disease severity may occur despite values remaining within published reference intervals. Concepts such as biological variation, subject-based reference values, and reference change values have therefore become increasingly important for the biological interpretation of laboratory data in both human and veterinary medicine [46,47,48].

Within this framework, the present study aimed to characterize systemic biomarker profiles associated with the clinical expression of SINS under commercial conditions rather than to identify diagnostic thresholds for individual biomarkers.

The present study identified distinct systemic biomarker profiles associated with the clinical expression of SINS in two farm cooperatives within the same commercial production system. Although cooperative 2 exhibited a higher overall prevalence of SINS, biomarker associations were generally stronger in cooperative 1, indicating that systemic inflammatory and metabolic responses are influenced not only by the clinical expression of SINS but also by farm-specific environmental and management conditions. While PigMAP emerged as the biomarker most consistently associated with the clinical SINS phenotype, associations with other hematological and clinical-chemical parameters differed between the two cooperatives. These findings are consistent with the concept that the systemic expression of SINS is modulated by environmental and management-related factors. The biological implications of these observations are discussed below. Pig major acute phase protein (PigMAP), also referred to as inter-alpha-trypsin inhibitor heavy chain 4 (ITIH4), is one of the major positive acute phase proteins in pigs [28,30,36,44,49,50]. PigMAP is not a cell-type-specific marker but a sensitive systemic biomarker reflecting generalized inflammatory activation [42,51]. In the present study, PigMAP showed positive associations with tail base bristle loss and teat reddening, whereas coronary band exudation, heel swelling, and tail tip reddening were negatively associated or showed no association. This pattern is consistent with the role of PigMAP as a marker of systemic inflammatory activity rather than secondary mechanical tissue damage [33,36,42,52,53]. Furthermore, the observed associations are difficult to reconcile with a predominantly mechanical etiology. If tail biting or technopathies were the principal drivers, the strongest associations would be expected with traumatic lesions rather than with the clinical manifestations identified in the present study. These findings are consistent with the endogenous SINS model, which has been supported by genetic predisposition studies [2,4,17,38,54,55], and histopathological observations of vasculitis, intimal proliferation, and thrombosis with macrophage and lymphocyte infiltration in intact epidermis of very young piglets [2]. PigMAP is primarily synthesized in the liver by hepatocytes, which respond to pro-inflammatory stimuli [32]. Primary triggers include tissue damage (DAMPs [danger associated molecular patterns]), lipopolysaccharides and similar MAMPs (microbe associated molecular patterns), as well as infections. This has been demonstrated directly in primary cultured porcine hepatocytes: stimulation with interleukin-6 (IL-6) or lipopolysaccharide (LPS) markedly increases both PigMAP mRNA expression and protein secretion, whereas other cytokines, such as TNF-α or IL-1, have comparatively smaller effects. Notably, IL-6 was shown to be upregulated in the liver of piglets affected by SINS [21]. The gene encoding PigMAP, ITIH4, is located on chromosome 13 and has previously been identified as a candidate gene associated with SINS [38].

Haptoglobin was identified as a potential predictor during the LASSO regression screening but did not reach statistical significance in the subsequent generalized linear models. This is consistent with the known kinetic profile of haptoglobin, which responds more slowly to inflammatory stimuli and is influenced by factors such as age, stress, and hemolysis [30,42]. In young pigs, haptoglobin concentrations may be insufficiently differentiated to reliably reflect subtle inflammatory activity. In contrast, C-reactive protein (CRP) has repeatedly been reported to be associated with SINS in previous studies [21,23], although CRP measurements were not available in the present study. Taken together, these findings indicate that PigMAP was the acute phase protein most strongly associated with the clinical expression of SINS in the present study, whereas haptoglobin appeared to be less informative under the conditions investigated.

### 4.2. Biological Interpretation of Clinical Chemistry Parameters

The clinical chemistry results can be interpreted along a biological gradient from functional, systemic markers to structural, cell-derived markers. Functional systemic markers, including creatinine and urea, primarily reflect protein turnover, muscle metabolism, and hydration status. In Farm 1, characterized by milder clinical expression of SINS and more favorable environmental conditions, both parameters were slightly decreased, suggesting a mild metabolic adaptation without evidence of overt catabolism [40,41,56]. In contrast, Farm 2, characterized by less favorable housing and health conditions together with a higher prevalence of SINS, showed relatively little variation in creatinine and urea concentrations among animals at the time of sampling. These findings may reflect physiological adaptation to persistent environmental and health-related stressors.

Integrative hepatic and metabolic markers, such as bilirubin, reflect hepatic uptake, erythrocyte turnover, and systemic metabolism rather than direct inflammatory tissue injury. The slightly lower bilirubin concentrations observed in Farm 2 are consistent with stress- or inflammation-associated metabolic adaptation rather than overt hepatocellular injury [57,58].

Cellular enzyme markers such as CK, LDH, and GLDH are typically released following structural cellular injury. In the present study, these enzyme activities were slightly lower in Farm 2 and remained unchanged in Farm 1. This pattern is consistent with metabolic adaptation and altered cellular metabolism rather than overt tissue necrosis [40,42,51,53,56].

Taken together, this hierarchical pattern is consistent with the concept that the systemic manifestations of SINS are primarily characterized by inflammatory and metabolic adaptations rather than overt organ or muscle necrosis. Platelet counts were lower in both herds, reaching statistical significance in the herd with less favorable environmental conditions (farm 2). This aligns with current understanding of thrombo-inflammation, where systemic inflammation modulates platelet number and function [59,60]. Reduced platelet counts may reflect endothelial activation or damage, consistent with the histological findings of vasculitis and thromboses [2,22]. However, reduced platelet counts are not specific for endothelial injury and may also occur in the context of concurrent inflammatory or infectious processes. Therefore, the present findings should be interpreted as being consistent with, rather than proving, endothelial involvement in SINS. Together with the observed PigMAP associations, these findings are consistent with the concept that the clinical expression of SINS is accompanied by systemic inflammatory responses and distinct systemic biomarker profiles. Inflammation-associated reductions in platelet counts are not unique to SINS. Experimental studies in pigs have shown that systemic inflammatory activation may contribute to platelet activation, consumption, and macrophage-mediated clearance, resulting in reduced platelet counts [61,62,63]. These findings are consistent with the interpretation that the reduced platelet counts observed in association with SINS reflect systemic inflammatory and hematological responses rather than localized mechanical injury. Serum calcium concentrations remained within the physiological range (2.7–2.8 mmol/L). Mild physiological variation in calcium homeostasis has previously been described during systemic inflammatory responses and is compatible with acute phase-associated metabolic adaptation rather than direct inflammatory tissue injury [64,65].

The study by Loewenstein et al. [22] documented substantially higher SINS scores than those observed in the present cohort. Systemic inflammatory activation was pronounced, as reflected by increased monocyte and neutrophil counts, elevated acute phase proteins such as CRP and fibrinogen, and moderate alterations in liver enzymes and metabolites (AST, total protein/globulins). Additionally, haemostatic changes, including reduced platelet counts, were detected, indicating involvement of the coagulation system. These findings illustrate that severe SINS is associated with widespread systemic effects, involving both the immune system and metabolic homeostasis.

By contrast, in the present study, SINS signs were milder, lesions were less extensive, and most hematological and clinical chemistry parameters showed only subtle changes. These differences may explain why many of the stronger associations between systemic biomarkers and lesion severity reported by Loewenstein et al. [22] were not observed in the present study. Differences in genetic background, growth performance, and body composition (slow-growing Parma Ham boars [66] versus extreme high-susceptibility boars), as well as the less pronounced inflammatory burden, may have contributed to the differences in biomarker magnitude between studies. However, the relative contributions of genetic background and clinical SINS severity cannot be disentangled in the present dataset because these factors were not varied independently. Taken together, these comparisons suggest that more pronounced clinical expression of SINS is accompanied by more extensive systemic inflammatory and metabolic alterations, whereas milder clinical manifestations are associated with more subtle systemic biomarker changes. Within this context, PigMAP emerged as the acute phase protein most consistently associated with the clinical expression of SINS in the present study, suggesting its potential value as a systemic biomarker for characterizing the syndrome under commercial production conditions.

### 4.3. Integration and Conclusions

The combined interpretation of acute phase proteins, clinical chemistry, and platelet counts is consistent with the concept that (i) PigMAP emerged as the acute phase protein most consistently associated with the clinical expression of SINS, whereas haptoglobin showed weaker associations, (ii) functional and metabolic markers (creatinine, urea, bilirubin) are associated with metabolic adaptation, (iii) structural enzyme markers (CK, LDH, GLDH) remain largely unchanged in the absence of extensive tissue damage, (iv) environmental and management-related factors may modulate the magnitude of the systemic response, and (v) reduced platelet counts may represent a secondary consequence of systemic inflammation. Although haptoglobin showed weak predictive signals in the LASSO screening, these associations were not retained in the subsequent GLM analyses. Under the field conditions investigated here, which were characterized by relatively mild SINS expression, PigMAP therefore appeared to be more informative than haptoglobin as an indicator of systemic inflammatory activity in young piglets. This finding should, however, be interpreted as exploratory rather than as evidence of superior diagnostic performance. Together, these findings provide a coherent biological characterization of the systemic biomarker profile associated with the clinical expression of SINS and support the concept of SINS as an endogenous systemic inflammatory syndrome rather than a purely mechanical disorder. At the same time, the present associations do not exclude interactions between endogenous susceptibility and external husbandry-related stressors, which may modulate the severity and clinical expression of SINS.

### 4.4. Herd-Specific Metabolic Adaptations Associated with Systemic Inflammation

The herd-specific biochemical patterns observed in the present study are most consistent with metabolic adaptations associated with systemic inflammation rather than uniform organ dysfunction. Clinical chemistry parameters reflect different physiological processes and therefore do not necessarily respond uniformly to inflammatory stimuli. In Farm Coop 1, characterized by more favorable housing and management conditions, the comparatively mild clinical expression of SINS was associated with slightly lower serum creatinine and urea concentrations, whereas bilirubin and intracellular enzyme activities (LDH, CK, and GLDH) remained unchanged. Creatinine and urea primarily reflect muscle metabolism, protein turnover, growth performance, hydration status, and feed intake rather than inflammatory tissue damage per se. Consequently, modest alterations in these parameters may reflect metabolic adaptation accompanying systemic inflammation rather than overt organ dysfunction [29,35,36].

The absence of appreciable changes in bilirubin and intracellular enzyme activities in Farm Coop 1 suggests preserved hepatic function and cellular integrity despite the presence of systemic inflammatory activity. This pattern is consistent with a mild inflammatory state in which metabolic regulation is altered without evidence of substantial tissue injury or cellular leakage [29,32].

In contrast, Farm Coop 2 was characterized by less favorable environmental conditions and a higher prevalence of SINS [5]. In this herd, SINS was associated with lower serum concentrations of bilirubin, LDH, CK, and GLDH, whereas creatinine and urea remained largely unchanged. This biochemical pattern is consistent with metabolic adaptation associated with sustained inflammatory and environmental stress rather than organ dysfunction.

Bilirubin reflects hepatic uptake, conjugation, and systemic metabolism rather than direct hepatocellular injury. Reduced bilirubin concentrations under prolonged inflammatory and stress conditions have been described as part of metabolic reallocation and altered hepatic transport activity rather than impaired liver function [39,40]. Similarly, LDH, CK, and GLDH are intracellular enzymes whose serum activities depend not only on tissue damage but also on metabolic activity, cellular turnover, and enzyme release kinetics. Previous studies have suggested that persistent inflammatory conditions may be accompanied by reduced metabolic activity and lower circulating enzyme activities, consistent with inflammation-associated hypometabolism [35,38].

The stability of creatinine and urea in Farm Coop 2 suggests a metabolic response that differed from that observed in Farm Coop 1. Whereas these functional markers were associated with SINS under the comparatively milder clinical conditions of Farm Coop 1, bilirubin and intracellular enzymes showed stronger associations in Farm Coop 2. Together, these findings are consistent with the involvement of different metabolic responses under different environmental conditions and levels of clinical expression.

Importantly, the observed parameter-specific changes do not indicate generalized organ dysfunction. A simultaneous reduction in functional markers (creatinine and urea), integrative hepatic markers (bilirubin), and intracellular enzymes (LDH, CK, and GLDH) might be expected in severe hepatic or muscular failure, cachexia, or systemic collapse, none of which was supported by the clinical presentation. Instead, the selective and herd-specific nature of the biochemical alterations is consistent with distinct systemic inflammatory and metabolic responses accompanying the clinical expression of SINS under different husbandry conditions.

### 4.5. Biochemical Patterns Associated with the Clinical Expression of SINS

The heterogeneous biochemical responses observed in the present study are consistent with differential metabolic adaptation accompanying systemic inflammation rather than generalized systemic deterioration. Comparable non-uniform biochemical responses have been described in chronic inflammatory conditions, where metabolic regulation may be modified without necessarily causing overt organ dysfunction [29,32]. Importantly, most biochemical parameters remained within established physiological reference ranges. However, this does not diminish their biological relevance. Clinical reference intervals are primarily intended to identify overt disease in individual animals, whereas the objective of the present study was to characterize biomarker profiles associated with the clinical expression of SINS. Consequently, systematic differences within physiological reference ranges may still reflect subtle inflammatory and metabolic adaptations associated with SINS and therefore provide valuable information relevant to animal health and welfare.

Together, the selective rather than uniform biochemical alterations complement the clinical phenotype and are consistent with the current concept of SINS as a condition involving systemic inflammation and metabolic adaptation, as proposed in previous histopathological and experimental studies [1]. The absence of parallel alterations across biochemical parameters argues against generalized hepatic or muscular pathology and is inconsistent with a disorder solely explained by localized mechanical lesions or toxin-mediated tissue injury. At the same time, the present associations cannot exclude interactions between endogenous susceptibility and external husbandry-related stressors, which may modulate the severity and clinical expression of SINS.

### 4.6. Limitations and Implications for Interpretation

The present study was designed to characterize systemic biomarker profiles associated with the clinical expression of SINS at weaning under commercial production conditions. Consequently, the results should be interpreted as associations between the clinical phenotype and systemic biomarker profiles rather than as evidence for the temporal sequence or causal relationships between individual clinical manifestations and subsequent inflammatory or metabolic responses. Addressing these questions will require longitudinal studies with repeated clinical examinations and serial blood sampling across production stages.

Previous studies have shown that the clinical expression of SINS differs between production stages, with some manifestations predominating during the suckling period whereas others persist into the nursery period [22]. The present study complements these observations by demonstrating that the clinical phenotype observed at weaning is accompanied by distinct hematological and biochemical biomarker profiles. Together, these findings are consistent with the concept of systemic inflammatory involvement in SINS while emphasizing that the temporal dynamics of these biomarker responses remain to be clarified.

Although multiple clinical chemistry parameters were analyzed, direct measurements of inflammatory mediators, such as cytokines or markers of endothelial activation, were not available. Consequently, systemic inflammatory activity could only be assessed indirectly through acute phase proteins and biochemical markers.

Furthermore, herd-specific differences in feeding, housing, management, health status, and infectious status likely contributed to the observed biochemical patterns. Consequently, differences between the two cooperatives cannot be attributed to individual herd-related factors. However, the principal comparisons between SINS scores and SINS status groups were performed within each cooperative. Thus, piglets compared within a cooperative were exposed to the same management conditions, health status, and infectious environment, thereby reducing the potential confounding effects of these herd-specific factors on the observed associations between SINS and biomarker profiles.

Despite these limitations, the consistent biological patterns observed across two independent commercial herds are consistent with the interpretation that the identified biomarker profiles are associated with the clinical expression of SINS rather than representing random analytical variation.

### 4.7. Implications for Future Research

Future studies should combine longitudinal clinical examinations with repeated hematological, biochemical, cytokine, and endothelial biomarker assessments to clarify the temporal relationships between clinical manifestations and systemic inflammatory responses and to identify biomarkers with potential relevance for herd-level monitoring.

## 5. Conclusions

The present study provides a biological characterization of the systemic biomarker profile associated with the clinical expression of Swine Inflammation and Necrosis Syndrome (SINS) under commercial production conditions. Using a combination of LASSO regression and generalized linear models, Pig major acute phase protein (PigMAP) emerged as the biomarker most consistently associated with the clinical SINS phenotype, while associations with creatinine, urea, calcium, bilirubin, and platelet counts further indicate that SINS is accompanied by measurable systemic inflammatory and metabolic alterations.

The observed biomarker profiles differed between the two commercial farm cooperatives, suggesting that the systemic expression of SINS is influenced not only by the clinical expression of the syndrome but also by herd-specific environmental and management conditions. Together with previous experimental, histopathological, and genetic studies, the present findings are consistent with the current concept of SINS as an endogenous systemic inflammatory condition rather than a disorder solely explained by localized mechanical lesions.

Integrating clinical lesion scoring with systemic biomarker profiling provides a broader biological understanding of SINS and establishes a foundation for future longitudinal studies combining repeated clinical examinations with serial biomarker assessments to further clarify the temporal relationships between clinical manifestations and systemic inflammatory responses.

From a practical perspective, the biomarkers investigated here should currently be regarded as research tools for characterizing systemic processes associated with SINS rather than as standalone diagnostic markers for individual animals. Their potential diagnostic application under field conditions requires further validation, particularly because biomarker responses may vary between herds and validated diagnostic thresholds for SINS are currently lacking.

## Figures and Tables

**Table 1:** Neonatal and weaned piglets’ clinical assessment.

Body Part	Sign	Description
Tail base	Hairless	Less than normal or no hair visible
	Redness	Redness of the skin is visible
	Swelling	Palpable subcutaneous oedema
	Exudate	Inflammatory liquid visible at the surface
	Necrosis	Necrotic tissue visible at the surface
	Bleeding	Blood is visible
Tail tip	Redness	Redness of the skin is visible
	Swelling	Palpable subcutaneous oedema
	Exudate	Inflammatory liquid is visible at the surface
	Necrosis	Necrotic tissue is visible at the surface
	Bleeding	Blood is visible
Ear basis	Hairless	Less than normal or no hair is visible
	Redness	Redness of the skin is visible
	Exudate	Inflammatory liquid visible at the surface
Ear tip	Redness	Redness of the skin is visible
Coronary band	Redness	Redness of the skin is visible
	Exudate	Inflammatory liquid is visible at the surface
	Swelling	Swollen tissue is visible
	Subcutaneous bleeding	Blood is visible
Teats	Redness	Redness of the teats is visible
	Swelling	Swollen tissue is visible
	Necrosis	Necrotic teats (black, dry) are visible
Heels	Swelling	Swollen tissue is visible
	Subcutaneous Bleeding	Blood is visible beneath the skin surface

**Table 2:** Prevalence of inflammation and necrosis at different body parts in suckling piglets and weaners by cooperation.

	Suckling Piglets		Weaners	
	Coop 1 *n* = 24	Coop 2 *n* = 21	Coop 1 *n* = 24	Coop 2 *n* = 21
Tail base	30.8 ± 9.5	37.9 ± 13.4	33.3 ± 9.3	49.2 ± 13.1
Tail tip	6.7 ± 4.8	4.2 ± 6.8	0.0 ± 5.2	6.7 ± 7.3
Ear	3.3 ± 4.8	4.2 ± 6.8	3.3 ± 4.7	6.3 ± 6.6
Coronary bands	59.2 ± 10.3	51.7 ± 14.6	0.0 ± 0.0	0.0 ± 0.0
Heels	2.5 ± 6.1 *	53.7 ± 8.6 *	0.0 ± 0.0	0.0 ± 0.0
Teats	8.3 ± 8.4	15.8 ± 11.9	0.0 ± 0.0	0.0 ± 0.0
SINS score > 0	69.2 ± 8.9	60.0 ± 12.5	33.3 ± 8.8	53.3 ± 12.5

* values within one category are significantly different between coops (*p* ≤ 0.05).

**Table 3:** Clinical chemical and hematological parameters by cooperation.

	Coop 1 *n* = 24		Coop 2 *n* = 21		*p*	Reference Intervals/Limits *
	Mean ± SE	CI95L–CI95U	Mean ± SE	CI95L–CI95U		
ALP (IU/L)	273.7 ± 18.6	236.0–311.4	433.8 ± 21.1	391.0–476.6	<0.001	≤300
ALT (IU/L)	53.6 ± 5.4	42.6–64.6	66.1 ± 6.2	53.6–78.7	n.s.	≤103
AST (IU/L)	119.2 ± 17.0	84.8–153.6	93.1 ± 19.3	54.0–132.1	n.s.	≤125
GLDH (IU/L)	3.4 ± 0.3	2.8–4.0	4.0 ± 0.3	3.3–4.6	n.s.	≤8
LDH (IU/L)	874.9 ± 63.1	746.8–1002.9	810.5 ± 71.7	665.1–956.0	n.s.	≤1893
CK (IU/L)	1775.5 ± 458.5	845.5–2705.4	1128.6 ± 520.8	72.4–2184.9	n.s.	≤10,101
GGT (IU/L)	107.2 ± 7.3	92.4–121.9	54.7 ± 8.2	38.0–71.4	<0.001	≤82
Haptoglobin (g/L)	0.6 ± 0.1	0.4–0.9	0.9 ± 0.1	0.6–1.1	n.s.	0.1–0.5
PigMAP (mg/L)	0.9 ± 0.1	0.8–1.1	0.8 ± 0.1	0.7–1.0	n.s.	0.7–1.2
Albumin (g/L)	27.6 ± 0.7	26.3–29.0	27.7 ± 0.7	26.2–29.2	n.s.	19–29
Total Protein (g/L)	48.7 ± 0.9	46.9–50.4	49.4 ± 1.0	47.4–51.4	n.s.	49–67
Total bilirubin (µmol/L)	2.8 ± 0.2	2.3–3.2	2.6 ± 0.2	2.1–3.1	n.s.	≤1.0
Urea (mmol/L)	4.5 ± 0.3	3.8–5.1	3.0 ± 0.4	2.3–3.7	0.005	1.7–4.5
Creatinine (µmol/L)	84.7 ± 2.6	79.4–90.1	83.2 ± 3.0	77.1–89.3	n.s.	88–130
NEFA (mmol/L)	0.4 ± 0.0	0.3–0.4	0.3 ± 0.0	0.3–0.4	n.s.	≤1.0
Calcium (mmol/L)	2.8 ± 0.0	2.7–2.8	2.6 ± 0.0	2.5–2.6	<0.001	2.5–3.1
Inorganic Phosphate (mmol/L)	5.5 ± 0.1	5.3–5.8	5.6 ± 0.2	5.3–5.9	n.s.	2.8–4.3
Magnesium (mmol/L)	1.6 ± 0.0	1.5–1.6	1.6 ± 0.0	1.5–1.6	n.s.	0.9–1.2
Hematocrit (%)	36.9 ± 1.1	34.7–39.0	36.2 ± 1.2	33.8–38.7	n.s.	34–44
Erythrocytes (10^12^/L)	5.8 ± 0.2	5.5–6.1	5.9 ± 0.2	5.5–6.2	n.s.	6.4–8.4
Hemoglobin (g/dL)	9.9 ± 0.5	9.0–10.9	9.8 ± 0.5	8.8–10.9	n.s.	105–135
MCV (fL)	63.9 ± 1.2	61.5–66.3	62.0 ± 1.3	59.3–64.8	n.s.	49–59
MCH (pg)	17.6 ± 0.4	16.8–18.4	17.1 ± 0.5	16.2–18.0	n.s.	20–25
MCHC (g/L)	27.5 ± 0.3	26.9–28.1	27.5 ± 0.3	26.8–28.2	n.s.	287–325
WBC (10^9^/L)	21.3 ± 1.4	18.4–24.2	14.9 ± 1.6	11.6–18.2	0.007	15.6–38.9
Lymphocytes (10^9^/L)	6.4 ± 0.6	5.3–7.6	4.8 ± 0.6	3.5–6.1	n.s.	7.7–20.4
Lymphocytes (%)	30.4 ± 2.3	25.7–35.1	34.4 ± 2.6	29.1–39.8	n.s.	40–60
Neutrophils (10^9^/L)	12.6 ± 1.2	10.2–14.9	9.2 ± 1.3	6.5–11.8	n.s.	3–17.4
Neutrophils (%)	59.3 ± 3.0	53.2–65.3	59.2 ± 3.4	52.4–66.1	n.s.	35–50
Monocytes (10^9^/L)	0.7 ± 0.1	0.5–0.8	0.4 ± 0.1	0.2–0.6	0.048	0.6–3.4
Monocytes (%)	3.2 ± 0.3	2.5–3.9	3.1 ± 0.4	2.3–3.9	n.s.	2–8
Platelets (10^9^/L)	543.0 ± 48.7	444.3–641.7	451.1 ± 55.3	338.9–563.2	n.s.	211–887

* Reference intervals or upper reference limits refer to nursery-age pigs where available and were used for biological context only. Statistical analyses were based on continuous values [23].

**Table 4:** Linear multiple regression to assess the association between SINS score and clinical-chemical parameters by coop and piglets’ age, controlling for age and sex.

Coop	SINS Score	Clin-Chem. Parameter	Zero-Order-Corr.	Partial Corr.	Zero-Order R^2^	Partial R^2^	*p*
1	SP *	Calcium (mmol/L)	0.636	0.661	0.40	0.44	0.019
1	SP	Creatinine (µmol/L)	−0.724	−0.726	0.52	0.53	0.008
1	SP	PigMAP (mg/L)	0.707	0.716	0.50	0.51	0.009
1	SP	Urea (mmol/L)	−0.785	−0.779	0.62	0.61	0.003
1	WP	Creatinine (µmol/L)	−0.443	−0.589	0.20	0.35	0.043
1	WP	PigMAP (mg/L)	0.595	0.612	0.35	0.37	0.035
2	SP	Total bilirubin (µmol/L)	−0.704	−0.749	0.50	0.56	<0.001
2	SP	CK (IU/L)	−0.471	−0.468	0.22	0.22	0.050
2	SP	GLDH (IU/L)	−0.532	−0.582	0.28	0.34	0.011
2	SP	Haptoglobin (g/L)	0.464	0.462	0.22	0.21	0.053
2	SP	LDH (IU/L)	−0.460	−0.461	0.21	0.21	0.054
2	WP	Total bilirubin (µmol/L)	−0.480	−0.493	0.23	0.24	0.038
2	WP	Calcium (mmol/L)	−0.543	−0.569	0.29	0.32	0.014

* SP = suckling piglets, WP = weaned piglets; R^2^: coefficient of determination; *p*: significance.

**Table 5:** Linear multiple regression to assess the association between tail base and clinical-chemical parameters by coop and piglets’ age, controlling for age and sex.

Coop	Tail Base Score	Clin-Chem. Parameter	Zero-Order-Corr.	Partial Corr.	Zero-Order R^2^	Partial R^2^	*p*
1	SP	Calcium (mmol/L)	0.581	0.579	0.34	0.33	0.049
1	SP	Creatinine (µmol/L)	−0.633	−0.722	0.40	0.52	0.008
1	SP	PigMAP (mg/L)	0.723	0.725	0.52	0.53	0.008
1	SP	Urea (mmol/L)	−0.689	−0.712	0.47	0.51	0.009
1	WP	Creatinine (µmol/L)	−0.443	−0.589	0.20	0.35	0.044
1	WP	PigMAP (mg/L)	0.595	0.612	0.35	0.37	0.035
2	SP	PigMAP (mg/L)	0.522	0.511	0.27	0.26	0.030
2	SP	Total bilirubin (µmol/L)	−0.533	−0.534	0.28	0.28	0.023
2	WP	Total bilirubin (µmol/L)	−0.472	−0.474	0.22	0.22	0.047

SP = suckling piglets, WP = weaned piglets; R^2^: coefficient of determination; *p*: significance.

**Table 6:** Clinical-chemical and hematological parameters by coop and SINS status.

	Coop1				Coop2			
SINS Status	0	1	2	*p*	0	1	2	*p*
ALP (IU/L)	258.0 ± 25.3 a	321.9 ± 20.0 b	245.3 ± 23.7 ab	≤0.05	495.0 ± 79.3	396.5 ± 79.3	419.4 ± 30.0	n.s.
ALT (IU/L)	57.7 ± 5.0	54.5 ± 3.9	52.6 ± 4.6	n.s.	68.5 ± 28.3	59.5 ± 28.3	64.7 ± 10.7	n.s.
AST (IU/L)	140.0 ± 40.0	117.6 ± 31.6	98.6 ± 37.4	n.s.	102.5 ± 39.0	82.5 ± 39.0	94.9 ± 14.7	n.s.
GLDH (IU/L)	4.3 ± 0.5	3.2 ± 0.4	3.4 ± 0.5	n.s.	5.2 ± 0.9	3.7 ± 0.9	3.7 ± 0.4	n.s.
LDH (IU/L)	1111.2 ± 138.3	818.1 ± 109.4	800.4 ± 129.4	n.s.	835.5 ± 148.8	732.0 ± 148.8	787.9 ± 56.2	n.s.
GGT (IU/L)	119.7 ± 16.4	93.2 ± 13.0	113.0 ± 15.4	n.s.	63.5 ± 14.6	48.5 ± 14.6	54.7 ± 5.5	n.s.
CK (IU/L)	2131.0 ± 1154.3	2052.6 ± 912.6	1078.8 ± 1079.8	n.s.	1552.0 ± 601.6	931.0 ± 601.6	1093.9 ± 227.4	n.s.
Haptoglobin (g/L)	0.6 ± 0.1	0.6 ± 0.1	0.7 ± 0.1	n.s.	0.5 ± 0.5	0.3 ± 0.5	1.0 ± 0.2	n.s.
PigMAP (mg/L)	0.8 ± 0.1 a	0.8 ± 0.1 a	1.1 ± 0.1 b	≤0.05	0.7 ± 0.2	0.9 ± 0.2	0.9 ± 0.1	n.s.
Albumin (g/L)	26.2 ± 1.3	28.2 ± 1.0	27.5 ± 1.2	n.s.	27.0 ± 1.9	31.5 ± 1.9	27.5 ± 0.7	n.s.
Total Protein (g/L)	50.3 ± 1.5	49.3 ± 1.1	48.3 ± 1.4	n.s.	47.5 ± 3.9	50.5 ± 3.9	48.6 ± 1.5	n.s.
Total bilirubin (µmol/L)	2.9 ± 0.4	2.6 ± 0.4	2.8 ± 0.4	n.s.	4.1 ± 0.4 a	2.1 ± 0.4 b	2.5 ± 0.1 b	≤0.01
Urea (mmol/L)	6.0 ± 0.7 a	4.1 ± 0.5 ab	3.5 ± 0.6 b	≤0.05	1.6 ± 0.8	2.2 ± 0.8	2.8 ± 0.3	n.s.
Creatinine (µmol/L)	96.5 ± 4.0	80.8 ± 3.1	82.5 ± 3.7	≤0.05	79.5 ± 10.3	77.5 ± 10.3	83.4 ± 3.9	n.s.
NEFA (mmol/L)	0.3 ± 0.0	0.3 ± 0.0	0.4 ± 0.0	n.s.	0.3 ± 0.1	0.3 ± 0.1	0.3 ± 0.0	n.s.
Calcium (mmol/L)	2.7 ± 0.1 a	2.8 ± 0.0 b	2.8 ± 0.0 b	≤0.05	2.6 ± 0.1	2.7 ± 0.1	2.6 ± 0.0	n.s.
Total Phosphate (mmol/L)	5.6 ± 0.2	5.7 ± 0.2	5.4 ± 0.2	n.s.	5.2 ± 0.5	6.0 ± 0.5	5.5 ± 0.2	n.s.
Magnesium (mmol/L)	1.5 ± 0.1	1.7 ± 0.1	1.5 ± 0.1	n.s.	1.6 ± 0.1	1.6 ± 0.1	1.5 ± 0.0	n.s.
Hematocrit (%)	37.9 ± 1.4	36.0 ± 1.1	36.7 ± 1.3	n.s.	40.4 ± 4.8	38.0 ± 4.8	35.6 ± 1.8	n.s.
Erythrocytes (10^12^/L)	6.1 ± 0.3	5.7 ± 0.2	5.7 ± 0.3	n.s.	5.9 ± 0.6	6.1 ± 0.6	5.8 ± 0.2	n.s.
Hemoglobin (g/dL)	10.6 ± 1.0	9.0 ± 0.8	10.2 ± 0.9	n.s.	11.1 ± 1.5	10.4 ± 1.5	9.8 ± 0.5	n.s.
MCV (fL)	62.4 ± 2.0	63.3 ± 1.6	65.0 ± 1.9	n.s.	67.9 ± 4.8	62.0 ± 4.8	61.7 ± 1.8	n.s.
MCH (pg)	17.3 ± 0.6	17.1 ± 0.5	18.0 ± 0.6	n.s.	18.6 ± 1.7	16.9 ± 1.7	17.1 ± 0.7	n.s.
MCHC (g/dL)	27.8 ± 0.5	27.0 ± 0.4	27.8 ± 0.5	n.s.	27.4 ± 1.1	27.3 ± 1.1	27.6 ± 0.4	n.s.
White blood cells (10^9^/L)	26.6 ± 3.2	22.7 ± 2.6	17.7 ± 3.0	n.s.	14.4 ± 3.8	12.1 ± 3.8	13.8 ± 1.4	n.s.
Lymphocytes (10^9^/L)	7.5 ± 1.2	7.3 ± 1.0	4.9 ± 1.2	n.s.	4.1 ± 1.3	5.1 ± 1.3	4.6 ± 0.5	n.s.
Lymphocytes (%)	29.0 ± 4.4	33.3 ± 3.5	27.3 ± 4.1	n.s.	29.0 ± 7.5	41.8 ± 7.5	34.6 ± 2.8	n.s.
Neutrophils (10^9^/L)	16.2 ± 2.8	13.2 ± 2.2	11.1 ± 2.6	n.s.	9.2 ± 2.9	5.6 ± 2.9	8.2 ± 1.1	n.s.
Neutrophils (%)	60.2 ± 6.3	57.5 ± 4.9	63.2 ± 5.9	n.s.	64.0 ± 8.2	46.4 ± 8.2	59.0 ± 3.1	n.s.
Monocytes (10^9^/L)	0.6 ± 0.2	0.7 ± 0.2	0.7 ± 0.2	n.s.	0.5 ± 0.1	0.5 ± 0.1	0.4 ± 0.0	n.s.
Monocytes (%)	2.3 ± 0.7	3.2 ± 0.6	3.5 ± 0.7	n.s.	3.2 ± 0.9	4.2 ± 0.9	3.1 ± 0.3	n.s.
Platelets (10^9^/L)	776.6 ± 103.0	553.7 ± 59.0	532.9 ± 101.7	n.s.	698.9 ± 132.4 a	376.1 ± 153.8 ab	231.4 ± 72.8 b	0.085 *

SP = suckling piglets, WP = weaned piglets; R2: coefficient of determination; p: significance. SINS status = 0: piglet with SINS score = 0 as suckling piglet and as weaner; 1: piglet with SINS score > 0 as suckling piglet or as weaner; 2: piglet with SINS score > 0 as suckling piglet and as weaner; n.s.: not significant; values within a row with different letters differ significantly; * this association was not statistically significant.

## Data Availability

The datasets generated and analyzed during the current study are not publicly available due to ongoing analyses and planned follow-up publications, but are available from the corresponding author on reasonable request.

## Abbreviations

The following abbreviations are used in this manuscript:

ALP	Alcalic phosphatase
ALT	Alanin aminotransferase
AST	Aspartat aminotransferase
CK	Creatine kinase
GGT	Gamma Glutamyltransferase
GLDH	Glutamate dehydrogenase
LDH	Lactate dehydrogenase
NEFA	Non-esterified fatty acids
MCV	Mean Corpuscular Volume
MCH	Mean Corpuscular Hemoglobin
MCHC	Mean Corpuscular Hemoglobin Concentration
PigMAP	Pig major acute-phase protein
SINS	Swine Inflammation and Necrosis Syndrome
WBC	White Blood Cells

## References

[1] Reiner G., Kuehling J., Loewenstein F., Lechner M., Becker S. (2021). Swine Inflammation and Necrosis Syndrome (SINS). Animals.

[2] Kuehling J., Loewenstein F., Wenisch S., Kressin M., Herden C., Lechner M., Reiner G. (2021). An in-depth diagnostic exploration of an inflammation and necrosis syndrome in a population of newborn piglets. Animal.

[3] Becker S., Hindenlang K., Kuehling J., Lechner M., Reiner G. (2025). Inflammation and Necrosis Syndrome in Young Piglets—A Longitudinal Study. Vet. Sci..

[4] Becker S., Kochendoerfer E., Kuehling J., Gerhards K., Lechner M., Zinner S., Lautner M., Reiner G. (2025). Breed- and Line-Dependent Severity of Inflammation and Necrosis Syndrome in AI Boars, and the Related Risk of Inflammation and Necrosis in Their Progeny. Vet. Sci..

[5] Koenders-van Gog K., Wijnands T., Lechner M., Reiner G. (2026). Longitudinal Study: Swine Inflammation and Necrosis Syndrome in Suckling and Weaned Piglets Is Associated with Tail Length and Integrity in Slaughter Pigs. Animals.

[6] European Union (2009). Council Directive 2008/120/EC laying down minimum standards for the protection of pigs. OJL.

[7] Wallgren T., Lundeheim N., Wallenbeck A., Westin R., Gunnarsson S. (2019). Rearing Pigs with Intact Tails-Experiences and Practical Solutions in Sweden. Animals.

[8] Gomes A., Romeo C., Ghidini S., Vieira-Pinto M. (2022). The Relationship between Carcass Condemnations and Tail Lesion in Swine Considering Different Production Systems and Tail Lengths. Animals.

[9] Van Staaveren N., Teixeira D.L., Hanlon A., Boyle L.A. (2017). Pig carcass tail lesions: The influence of record keeping through an advisory service and the relationship with farm performance parameters. Animal.

[10] Vom Brocke A.L., Karnholz C., Madey-Rindermann D., Gauly M., Leeb C., Winckler C., Schrader L., Dippel S. (2019). Tail lesions in fattening pigs: Relationships with postmortem meat inspection and influence of a tail biting management tool. Animal.

[11] Gerster U., Sidler X., Wechsler B., Nathues C. (2022). Prevalence of tail lesions in Swiss finishing pigs. Schweiz. Arch. Tierheilkd..

[12] Valros A., Ahlström S., Rintala H., Häkkinen T., Saloniemi H. (2004). The prevalence of tail damage in slaughter pigs in Finland and associations to carcass condemnations. Acta Agric. Scand. Sect. A—Anim. Sci..

[13] Heinonen M., Välimäki E., Laakkonen A.M., Toppari I., Vugts J., Fàbrega E., Valros A. (2021). Evaluation of Tail Lesions of Finishing Pigs at the Slaughterhouse: Associations With Herd-Level Observations. Front. Vet. Sci..

[14] Valros A. (2022). Review: The tale of the Finnish pig tail—How to manage non-docked pigs?. Animal.

[15] Teixeira D.L., Harley S., Hanlon A., O’Connell N.E., More S.J., Manzanilla E.G., Boyle L.A. (2016). Study on the Association between Tail Lesion Score, Cold Carcass Weight, and Viscera Condemnations in Slaughter Pigs. Front. Vet. Sci..

[16] Kongsted H., Foldager L., Sørensen J.T. (2020). Data from routine meat inspection is a poor indicator of the prevalence of tail lesions in undocked pigs. Porc. Health Manag..

[17] Leite N.G., Knol E.F., Nuphaus S., Vogelzang R., Tsuruta S., Wittmann M., Lourenco D. (2023). The genetic basis of swine inflammation and necrosis syndrome and its genetic association with post-weaning skin damage and production traits. J. Anim. Sci..

[18] Micout S., Fortune H., Reiner G. (2025). Prevalence and Consequences of Swine Inflammation and Necrosis Syndrome (SINS) in French Herds. Vet. Sci..

[19] Koenders-van Gog K., Wijnands T., Lechner M., Reiner G., Fink-Gremmels J. (2025). Screening of Piglets for Signs of Inflammation and Necrosis as Early Life Indicators of Animal Health and Welfare Hazards. Animals.

[20] Reiner G., Kuehling J., Lechner M., Schrade H.J., Saltzmann J., Muelling C., Daenicke S., Loewenstein F. (2020). Swine Inflammation and Necrosis Syndrome is influenced by husbandry and quality of sow in suckling piglets, weaners and fattening pigs. Porc. Health Manag..

[21] Ringseis R., Gessner D., Löwenstein F., Kuehling J., Becker S., Willems H., Lechner M., Eder K., Reiner G. (2021). Swine inflammation and necrosis syndrome is associated with plasma metabolites and liver transcriptome in affected piglets. Animals.

[22] Loewenstein F., Becker S., Kuehling J., Schrade H., Lechner M., Ringseis R., Eder K., Moritz A., Reiner G. (2022). Inflammation and necrosis syndrome is associated with alterations in blood and metabolism in pigs. BMC Vet. Res..

[23] Gerhards K., Becker S., Kuehling J., Lechner M., Willems H., Ringseis R., Reiner G. (2025). Screening for transcriptomic associations with Swine Inflammation and Necrosis Syndrome. BMC Vet. Res..

[24] Heinrich P.C., Castell J.V., Andus T. (1990). Interleukin-6 and the acute phase response. Biochem. J..

[25] Dinarello C.A. (2000). Proinflammatory cytokines. Chest.

[26] Baumann H., Gauldie J. (1994). The acute phase response. Immunol. Today.

[27] Cray C., Zaias J., Altman N.H. (2009). Acute phase response in animals: A review. Comp. Med..

[28] Petersen H.H., Nielsen J.P., Heegaard P.M.H. (2004). Application of acute phase protein measurements in veterinary clinical chemistry. Vet. Res..

[29] Gruys E., Toussaint M.J.M., Niewold T.A., Koopmans S.J. (2005). Acute phase reaction and acute phase proteins. J. Zhejiang Univ. Sci. B.

[30] Heegaard P.M.H., Klausen J., Nielsen J.P., González-Ramón N., Piñeiro M., Lampreave F., Alava M.A. (2011). The porcine acute phase response to infection: A review. Vet. Immunol. Immunopathol..

[31] Heegaard P.M.H., Klausen J., Nielsen J.P., González-Ramón N., Piñeiro M., Lampreave F., Alava M.A. (1998). The porcine acute phase response to infection with *Actinobacillus pleuropneumoniae*: Haptoglobin, C reactive protein, major acute phase protein and serum amyloid A protein are sensitive indicators of infection. Comp. Biochem. Physiol. B.

[32] Skovgaard K., Mortensen S., Boye M., Poulsen K.T., Campbell F.M., Eckersall P.D., Heegaard P.M. (2009). Rapid and widely disseminated acute phase protein response after experimental bacterial infection of pigs. Vet. Res..

[33] Pomorska-Mól M., Markowska-Daniel I., Kwit K., Pejsak Z. (2013). C-reactive protein, haptoglobin, serum amyloid A and Pig-MAP response in pigs coinfected with H1N1 swine influenza virus and *Pasteurella multocida*. BMC Vet. Res..

[34] Sorensen N.S., Tegtmeier C., Andresen L.O., Piñeiro M., Toussaint M.J., Campbell F.M., Lampreave F., Heegaard P.M. (2006). The porcine acute phase protein response to acute clinical and subclinical experimental infection with Streptococcus suis. Vet. Immunol. Immunopathol..

[35] Pomorska-Mól M., Markowska-Daniel I., Kwit K., Stępniewska K., Pejsak Z. (2015). Profile of the porcine acute-phase proteins response following experimental co-infection with H3N2 swine influenza virus and Pasteurella multocida. Biomarkers.

[36] González-Ramón N., Hoebe K., Alava M.A., van Leengoed L., Lampreave F., Piñeiro A. (2000). Pig MAP/ITIH4 and haptoglobin are interleukin-6-dependent acute-phase plasma proteins in porcine primary cultured hepatocytes. Eur. J. Biochem..

[37] Ma Y., Li R., Wang J., Jiang W., Yuan X., Cui J., Wang C. (2021). ITIH4, as an inflammation biomarker, mainly increases in bacterial bloodstream infection. J. Clin. Lab. Anal..

[38] Gerhards K., Becker S., Kuehling J., Lechner M., Bathke J., Willems H., Reiner G. (2023). GWAS reveals genomic associations with swine inflammation and necrosis syndrome. Mamm. Genome.

[39] (2015). Quality Management Systems—Requirements.

[40] Kaneko J.J., Harvey J.W., Bruss M.L. (2008). Clinical Biochemistry of Domestic Animals.

[41] Stockham S.L., Scott M.A. (2008). Fundamentals of Veterinary Clinical Pathology.

[42] Eckersall P.D., Bell R. (2010). Acute phase proteins: Biomarkers of infection and inflammation in veterinary medicine. Vet. J..

[43] Schumann G., Bonora R., Ceriotti F., Clerc-Renaud P., Ferrero C.A., Férard G., Franck P.F.H., Gella F.J., Hoelzel W., Jørgensen P.J. (2002). IFCC primary reference procedures for the measurement of catalytic activity concentrations of enzymes at 37 °C. Part 1: General considerations. Clin. Chem. Lab. Med..

[44] Robinson N.A., Loynachan A.T., Zimmerman J.J., Karriker L.A., Ramirez A., Schwartz K.J., Stevenson G.W., Zhang J. (2019). Cardiovascular and hematopoietic systems. Diseases of Swine.

[45] Tibshirani R. (1996). Regression shrinkage and selection via the Lasso. J. R. Stat. Soc. Ser. B.

[46] Walton R.M. (2012). Subject-based reference values: Biological variation, individuality, and reference change values. Vet. Clin. Pathol..

[47] Friedrichs K.R., Harr K.E., Freeman K.P., Szladovits B., Walton R.M., Barnhart K.F., Blanco-Chavez J. (2012). ASVCP reference interval guidelines: Determination of de novo reference intervals in veterinary species and other related topics. Vet. Clin. Pathol..

[48] Badrick T. (2021). Biological variation: Understanding why it is so important?. Pract. Lab. Med..

[49] Piñeiro M., Piñeiro C., Carpintero R., Morales J., Campbell F.M., Eckersall P.D., Toussaint M.J.M., Lampreave F. (2007). Characterisation of the pig acute phase protein response to different diseases. Vet. Immunol. Immunopathol..

[50] Koene M.G.J., Mulder H.A., Stockhofe-Zurwieden N., Kruijt L., Smits M.A. (2012). Serum protein profiles as potential biomarkers for infectious disease status in pigs. BMC Vet. Res..

[51] Ceciliani F., Ceron J.J., Eckersall P.D., Sauerwein H. (2012). Acute phase proteins in ruminants. J. Proteom..

[52] Grau-Roma L., Heegaard P.M.H., Hjulsager C.K., Sibila M., Kristensen C.S., Allepuz A., Piñeiro M., Lampreave F., Fraile L., Segalés J. (2009). Pig-MAP and haptoglobin serum concentrations in porcine circovirus type 2 infection. Vet. J..

[53] Murata H., Shimada N., Yoshioka M. (2004). Current research on acute phase proteins in veterinary diagnosis: An overview. Vet. J..

[54] Kuehling J., Eisenhofer K., Lechner M., Becker S., Willems H., Reiner G. (2021). The effects of boar on susceptibility to swine inflammation and necrosis syndrome in piglets. Porc. Health Manag..

[55] Oliveira Garcia A., Galoro Leite N., Vogelzang R., Bretanha Rocha R.F., Facioni Guimarães S.E., Lourenco D. (2026). Genetic architecture of swine inflammation and necrosis syndrome and its genetic correlation with repeated skin damage scores. J. Anim. Sci..

[56] Tvedten H. (2017). Small Animal Clinical Diagnosis by Laboratory Methods.

[57] Smith B.P. (2009). Large Animal Internal Medicine.

[58] Hall E.J., Clarke D.D., Collins G. (2003). Biochemical monitoring of liver function in clinical practice. Vet. Rec..

[59] Semple J.W., Italiano J.E., Freedman J. (2011). Platelets and the immune continuum. Nat. Rev. Immunol..

[60] Stark K., Massberg S. (2021). Interplay between inflammation and thrombosis in cardiovascular pathology. Nat. Rev. Cardiol..

[61] Zaid Y., Merhi Y. (2022). Immunothrombosis and thromboinflammation in host defense and disease. Int. J. Mol. Sci..

[62] Halbur P.G., Paul P.S., Frey M.L., Landgraf J., Eernisse K., Meng X.-J., Lum M.A., Andrews J.J., Rathje J.A. (1995). Comparison of the pathogenicity of two U.S. porcine reproductive and respiratory syndrome virus isolates with that of the Lelystad virus. Vet. Pathol..

[63] Segalés J., Domingo M. (2002). Postweaning multisystemic wasting syndrome (PMWS) in pigs. A review. Vet. Q..

[64] Holowaychuk M.K., Martin L.G. (2007). Review of hypocalcemia in septic patients. J. Vet. Emerg. Crit. Care.

[65] Rodríguez Alarcón J., López González C., Márquez E., García López J. (2019). Cytokines and calcium homeostasis: Couriers of inflammation and adjustment mechanisms. Adv. Clin. Chem..

[66] Consorzio del Prosciutto di Parma. https://www.prosciuttodiparma.com/wp-content/uploads/Parma-Ham-Specification-FINAL-2.3.2022.pdf.

